# Human–Generative AI Interactions and Their Effects on Beliefs About Health Issues: Content Analysis and Experiment

**DOI:** 10.2196/80270

**Published:** 2026-02-04

**Authors:** Linqi Lu, Yanshu Sybil Wang, Jiawei Liu, Douglas M McLeod

**Affiliations:** 1 Department of Communication University of North Dakota Grand Forks, ND United States; 2 School of Journalism and Mass Communication University of Wisconsin–Madison Madison, WI United States; 3 STEM Translational Communication Center College of Journalism and Communications University of Florida Gainesville, FL United States; 4 Department of Advertising College of Journalism and Communications University of Florida Gainesville, FL United States

**Keywords:** generative AI, ChatGPT, human-AI interaction, flu vaccination, climate change, artificial intelligence

## Introduction

At the intersection of generative artificial intelligence (AI) and health issues, where misconceptions proliferate, the question remains: does generative AI improve public understanding of health issues? Health misconceptions stem from false or factually inaccurate information and a lack of health literacy [1]. For flu vaccination and climate change, where misconceptions are common and have behavioral and policy implications [2,3], addressing the problem represents an urgent need.

On the pessimistic side, generative AI technology may produce factually inaccurate content inadvertently, as generative AI tools are content generators, not necessarily fact generators. Content generation relies on training data and underlying algorithms, but if the data used include outdated information, generative AI tools may produce inaccurate information [4]. AI may also ignore inaccuracies in users’ content generation prompts or create content that is tailored to receiver preferences, which may reinforce existing misconceptions, resulting in echo chambers [5].

From an optimistic perspective, generative AI tools may be used to evaluate health information and improve public understanding. Companies are incentivized to validate the objectivity of their AI tools to legitimize them [6]. Harmful AI output may be diminished through supervised and reinforcement learning, and AI tools may reduce misperceptions among their users. In such cases, generative AI may help lessen health-related misconceptions.

Given the contradictory roles, this study investigated the content and effects of large language model–based human-AI interactions that evaluate information related to flu vaccination and climate change (including widespread myths). First, using GPT-4o to analyze human-ChatGPT conversations, we examined whether responses from ChatGPT engaged in any well-established communication strategies that were identified by existing meta-analyses to improve accurate understanding of health issues [7,8], including coherence appeals (providing explanations against misconceptions) [7], credibility appeals (highlighting official agencies’ statements) [7], consensus appeals (highlighting the agreement among experts) [7], verification appeals (encouraging users to cross-check information) [7], and empathy appeals (acknowledging users’ experiences/concerns) [9]. Second, we also examined whether user interactions with ChatGPT lead to changes in misconceptions and attitudes on issues.

## Methods

### Overview

Undergraduate students in communication courses from a large midwestern university in the United States were invited to use ChatGPT (GPT-3.5 or GPT-4, depending on whether the respondent used the free or paid version) to evaluate information (including widespread myths) related to flu vaccination and climate change in an online study in exchange for extra course credit. A total of 217 students accessed the study, with 149 students completing the questionnaire. We measured respondents’ misconceptions and attitudes on issues both before and after their interactions with ChatGPT (using items with 7-point scales; see Multimedia Appendix 1). Paired samples *t* tests were conducted to test the difference between the posttest and pretest measures. We also collected the transcripts of all user-ChatGPT interactions (149 respondents × 2 issues = 298 transcripts) and used both GPT-4o and human coding to analyze each transcript for the presence of the communication strategies in ChatGPT’s responses (any discrepancies between GPT-4o and the human coder were subsequently reviewed and validated by a second coder and the analyses were based on the verified dataset).

### Ethical Considerations

The study was approved by the institutional review board of the University of Wisconsin–Madison (IRB# 2023-1416), and informed consent was obtained from all participants. Participants took the study in exchange for extra course credit, and the data collected were anonymized/deidentified.

## Results

**Figure 1:**
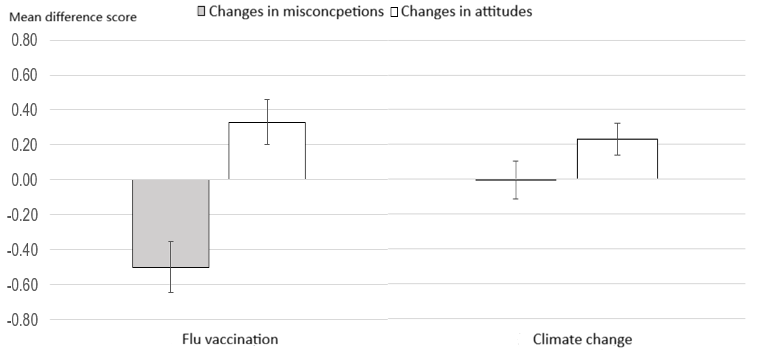
Changes in misconceptions and positive attitudes/support for flu vaccination and climate action (with 95% CIs).

Coherence appeals appeared in all 149 transcripts for both issues (n=149, 100%), followed by consensus appeals (n=65, 43.6% for flu vaccination and n=137, 91.9% for climate change), credibility appeals (n=58, 38.9% for flu vaccination and n=90, 60.4% for climate change), verification appeals (n=88, 59.1% for flu vaccination and n=14, 9.4% for climate change), and empathy appeals (n=77, 51.7% for flu vaccination and n=9, 6.0% for climate change; see Table 1). Interactions with ChatGPT were associated with lower misconceptions about flu vaccination (posttest mean 2.43, SD 1.24 compared with pretest mean 2.93, SD 1.13; *d*=–0.56; *P*<.001) but not climate change (posttest mean 2.20, SD 0.96 compared with pretest mean 2.20, SD 0.99; *d*=–0.01; *P*=.94). Conversations with ChatGPT were also associated with increased positive attitudes toward flu vaccination (posttest mean 6.18, SD 1.14 compared with pretest mean 5.84, SD 1.29; *d*=0.41; *P*<.001) and support for climate action (posttest mean 5.81, SD 1.02 compared with pretest mean 5.58, SD 1.08; *d*=0.41; *P*<.001; Figure 1). Exposure to information about the high versus low credibility of ChatGPT did not affect these outcomes (Multimedia Appendix 1).

**Table 1:** Variables in focus in the analysis of ChatGPT-generated content (N=149) for each issue topic.

Variable definition			Variable operationalization (representative quotes)		GPT-4o–assisted content analysis count, n (%)		Agreement between GPT-4o and human coder (%)	
**Coherence appeals: providing explanations to support statements against misconceptions [7]**								93
	Flu vaccination	“The flu vaccine is important for several reasons: Preventing Illness: The flu vaccine is designed to protect against the influenza viruses that are expected to be most common during the flu season. Getting vaccinated reduces your risk of getting sick with the flu. Reducing Severity of Illness: Even if you do get the flu after being vaccinated, the vaccine can still reduce the severity and duration of the illness. This is particularly important for vulnerable populations, such as the elderly and individuals with underlying health conditions...”		149 (100.0)				
	Climate change	“Here are some key reasons why climate change is a critical issue: Environmental Impact: It leads to rising temperatures, melting ice caps, sea level rise, disruptions in ecosystems, loss of biodiversity, and changes in weather patterns, affecting natural habitats and the balance of ecosystems. Human Health: Climate change contributes to increased frequency and intensity of extreme weather events, which can result in loss of lives, injuries, displacement of communities, and the spread of diseases...”		149 (100.0)				
**Consensus appeals: highlighting the overall agreement among experts [7]**								94
	Flu vaccination	“The general consensus among healthcare professionals and public health organizations is that flu vaccination is beneficial.”		65 (43.6)				
	Climate change	“The overwhelming scientific consensus is that the increased concentration of greenhouse gases from human activities, such as burning fossil fuels and deforestation, is the dominant factor driving the observed warming of the planet.”		137 (91.9)				
**Credibility appeals: highlighting official agencies’ statements/opinions [7]**								88
	Flu vaccination	“Public health organizations, such as the World Health Organization (WHO) and the Centers for Disease Control and Prevention (CDC), often advocate for widespread flu vaccination to help control the spread of the flu virus and protect vulnerable populations.”		58 (38.9)				
	Climate change	“The Intergovernmental Panel on Climate Change (IPCC) and numerous scientific organizations worldwide, such as the National Aeronautics and Space Administration (NASA) and the National Oceanic and Atmospheric Administration (NOAA), have stated that human activities are the primary driver of recent global warming.”		90 (60.4)				
**Verification appeals: motivating users to cross-check information [7]**								90
	Flu vaccination	“If you have concerns about the flu vaccine or experience unusual symptoms after vaccination, it’s always a good idea to consult with your healthcare provider for guidance based on your individual health situation.”		88 (59.1)				
	Climate change	“It’s essential to rely on accurate and up-to-date scientific information when discussing complex issues like climate change.”		14 (9.4)				
**Empathy appeals: acknowledging users’ experiences, feelings, or beliefs [9]**								90
	Flu vaccination	“It’s not uncommon for some people to experience mild symptoms after receiving the flu vaccine.”		77 (51.7)				
	Climate change	“Climate change continues to be a significant global concern.”		9 (6.0)				

## Discussion

Our research examines human–generative AI interactions across two health issues. Our content analysis of human-ChatGPT conversations revealed that ChatGPT used a variety of well-established strategies to improve accurate understanding of health issues [7-9]. Moreover, experimental findings showed that such conversations were associated with reduced misconceptions and increased support for actions. Despite several limitations (Multimedia Appendix 1), the findings indicate that the use of ChatGPT might be beneficial in boosting health literacy, and future research may expand our insights by looking into other issues and using a nationally representative sample.

## Appendix

Multimedia Appendix 1 Study design and measures, content analysis, and study limitations.

## Abbreviations

AI: artificial intelligence

## Data Availability

The dataset is available from the corresponding author upon reasonable request.
