# Supplementary material for: Human–Generative AI Interactions and Their Effects on Beliefs About Health Issues: Content Analysis and Experiment
Source: JMIR AI. 2026 Feb 4;5:e80270. doi: 10.2196/80270 (PMC12917482; doi:10.2196/80270)
Supplement: Multimedia Appendix 1 [file ai_v5i1e80270_app1.docx]

**Appendix. Supplemental Materials**

**Study Design and Measurements**

This study adopted a pretest-posttest design as a pretest-posttest design provided us with more statistical power with the same sample size than a posttest-only control group design. We measured respondents’ misconceptions and issue attitudes toward flu vaccination and climate change both before and after their interactions with ChatGPT. The order of topic/issue was randomized. With the pretest-posttest design, paired samples t-tests were performed comparing posttest results to pretest results. For a paired sample t-test, to detect a small to moderate effect size of *d_z_* = .30 with α = .05 and statistical power of .80, we need a sample size of 90. Our final sample size of 149 well satisfied this requirement. Among these 149 undergraduate students in communication courses from a large midwestern university, the average age was 20.87 (SD = 1.14). 79.2% were female and 20.8% were male. 77.2% were White, 2.7% were Black or African American, 16.1% were Asian, and 3.4% identified with more than one racial group. Regarding ethnicity, 6% identified as Hispanic or Latino/a/x. The specific measures were listed below.

**Flu Vaccination**

**Misconceptions about Flu Vaccination** (false or inaccurate beliefs about flu vaccination that one holds: measured both before and after human-ChatGPT interaction): Each item was measured on a 7-point scale from 1 = strongly disagree and 7 = strongly agree and the six items were averaged to form the misconceptions about flu vaccination index (M = 2.93, SD = 1.13, α = .81 for the pretest measure and M = 2.43, SD = 1.24, α = .89 for the posttest measure).

Please indicate your level of agreement with each of the following statements about flu vaccination. (Adapted from University of California San Francisco Health: <https://www.ucsfhealth.org/education/top-seven-flu-myths-debunked>)

The flu vaccine can give you the flu.

It’s no big deal to get the flu.

Young and healthy people do not need to get the flu vaccine.

People who get the flu vaccine can still get the flu, so it’s not worth getting vaccinated.

People don’t need to get the vaccine every year.

If a person has a chronic illness or is pregnant, they shouldn’t get the flu vaccine.

**Attitudes toward Flu Vaccination** (one’s valenced evaluation of flu vaccination: measured both before and after human-ChatGPT interaction): Each item was measured on a 7-point semantic differential scale and the four items were averaged to form the attitudes toward flu vaccination index (M = 5.84, SD = 1.29, α = .94 for the pretest measure and M = 6.18, SD = 1.14, α = .94 for the posttest measure).

Getting flu vaccination is ___________

|  | 1 | 2 | 3 | 4 | 5 | 6 | 7 |  |
| --- | --- | --- | --- | --- | --- | --- | --- | --- |
| Bad |  |  |  |  |  |  |  | Good |
| Undesirable |  |  |  |  |  |  |  | Desirable |
| Harmful |  |  |  |  |  |  |  | Beneficial |
| Foolish |  |  |  |  |  |  |  | Wise |

**Instructions for Human-ChatGPT Interaction**

Please discuss with ChatGPT about flu vaccination for 5 mins and paste the full script of your interactions (i.e., your queries and ChatGPT’s responses) in the textbox provided.

You will be asked to evaluate the following statements again after your interaction with ChatGPT, so please discuss with ChatGPT carefully. (The statements shown to the respondents here are the same as those statements used in measuring flu vaccination related misconceptions.)

Please do not refer to other sources during the interaction. You will be able to proceed to the next page after 5 mins but please feel free to use ChatGPT longer to prepare you to answer related questions.

**Climate Change**

**Misconceptions about Climate Change** (false or inaccurate beliefs about climate change that one holds: measured both before and after human-ChatGPT interaction): Each item was measured on a 7-point scale from 1 = strongly disagree and 7 = strongly agree. The third and fifth items were reverse coded and then the five items were averaged to form the misconceptions about climate change index (M = 2.20, SD = 0.99, α = .81 for the pretest measure and M = 2.20, SD = 0.96, α = .72 for the posttest measure).

Please indicate your level of agreement with each of the following statements about climate change. (Adapted based on the frequently asked questions about climate change identified by the National Aeronautics and Space Administration: <https://science.nasa.gov/climate-change/faq/>)

It’s the sun causing global warning [*sic*].

Carbon dioxide (CO2) doesn’t have significant impact on Earth’ [*sic*] climate.

Most scientists agree that human-caused climate change is happening.

Climate models that simulate past, present, and future climate conditions are not accurate.

Human activities are contributing significantly to climate change.

**Support for Climate Action** (measured both before and after human-ChatGPT interaction): Each item was measured on a 7-point scale. The second item was reverse coded and then the three items were averaged to form the support for climate action index (M = 5.58, SD = 1.08, α = .81 for the pretest measure and M = 5.81, SD = 1.02, α = .83 for the posttest measure).

How worried are you about climate change? (7- point scale from 1 = not worried at all to 7 = extremely worried)

The government is doing _____ to address climate change. (7- point scale from 1 = too little to 7 = too much)

Do you think the government should be doing more or less to address climate change? (7-point scale from 1 = much less to 7 = much more)

**Instructions for Human-ChatGPT Interaction**

Please discuss with ChatGPT about climate change for 5 mins and paste the full script of your interactions (i.e., your queries and ChatGPT’s responses) in the textbox provided.

You will be asked to evaluate the following statements again after your interaction with ChatGPT, so please discuss with ChatGPT carefully. (The statements shown to the respondents here are the same as those statements used in measuring climate change related misconceptions.)

Please do not refer to other sources during the interaction. You will be able to proceed to the next page after 5 mins but please feel free to use ChatGPT longer to prepare you to answer related questions.

*Note.* Before the measurements related to specific issues above, we also randomly exposed each respondent to information about either high vs. low credibility of ChatGPT assuming that such information would affect respondents’ perceived credibility of ChatGPT. Specifically, high ChatGPT credibility information shows that “Supporters of the use of ChatGPT argue that: ChatGPT operates out of users’ best interests. ChatGPT can provide accurate and fair information on various topics. ChatGPT protects user data from privacy and copyright related concerns. ChatGPT encourages diverse views on issues, amplifying different perspectives. Using ChatGPT in learning and education can improve critical thinking. ChatGPT often works well in completing the tasks based on user requests.” By comparison, low ChatGPT credibility information shows that “Opponents against the use of ChatGPT argue that: ChatGPT does not operate out of users’ best interests. ChatGPT may produce biased or inaccurate content. ChatGPT’s use of user data may cause privacy and copyright related concerns. ChatGPT might reinforce existing views on issues, amplifying echo chambers. Reliance on ChatGPT may reduce people’s ability to think critically. ChatGPT often fails to complete the tasks based on user requests.”

Perceived credibility was measured based on respondents’ agreement with 11 statements, each on a 7-point scale from 1 = strongly disagree to 7 = strongly agree, including: “ChatGPT cares about our well-being”; “ChatGPT is sincerely concerned about addressing the problems of human users”; “ChatGPT tries to be helpful and do [*sic*] not operate out of selfish interest”; “ChatGPT is truthful in its dealings”; “ChatGPT keeps its commitments and deliver [*sic*] on its promises”; “ChatGPT is honest and do [*sic*] not abuse the information and advantage it has over its users”; “ChatGPT works well”; “ChatGPT has the features necessary to complete key tasks”; “ChatGPT is reliable”; “ChatGPT is dependable”; and “ChatGPT is competent in its area of expertise”.

An independent sample t-test showed that the ChatGPT credibility information did not have a significant effect on perceived ChatGPT credibility (the mean difference was very small: M = 4.44, SD = 0.83 for the high credibility information vs. M = 4.39, SD = 1.07 for the low credibility information, *t*(139) = .32, *p* = .75). Thus, the manipulation check failed: there was no significant difference in perceived credibility between groups. Further analyses showed that neither did the manipulation significantly affect any changes in issue related misconceptions or attitudes (all *p*s > .05). Therefore, data from both credibility conditions were pooled/collapsed for the main analyses.

**Content Analysis Using GPT-4o to Detect the Presence of Specific Communication Features**

Model= GPT-4o

Word limit=1000

Temperature=0

# Background Knowledge

background_knowledge = """

You are tasked with detecting and analyzing human and chatbot conversations based on the following criteria:

- Coherence Appeals: Providing explanations to counter misinformation.

- Consensus Appeals: Highlighting expert agreement on a topic.

- Credibility Appeals: Referencing reputable institutions or sources.

- Empathy Appeals: Acknowledging the user’s emotions, experiences, or concerns.

- Encouragement to Verify: Motivating users to cross-check information with reliable sources.

Each interaction should be analyzed thoroughly for the presence of these features.

"""

# Classification Prompt Template

prompt_template = """

You are tasked with performing a multilabel classification of a conversation between a user and ChatGPT. Carefully analyze each interaction and assign appropriate labels based on the following categories:

### **Coherence Appeals**

- Does ChatGPT provide logical explanations to counter misinformation? (Yes/No)

- Example: "The flu vaccine is important because it helps prevent illness and reduces severity if infected."

### **Consensus Appeals**

- Does ChatGPT highlight expert agreement on the topic? (Yes/No)

- Example: "The scientific consensus is that human activities are the primary driver of climate change."

### **Credibility Appeals**

- Does ChatGPT cite reputable organizations or experts? (Yes/No)

- Example: "According to the CDC and WHO, flu vaccines are recommended for public health."

### **Empathy Appeals**

- Does ChatGPT acknowledge or validate user concerns? (Yes/No)

- Example: "It's understandable that you might have concerns about the flu vaccine."

### **Encouragement to Verify**

- Does ChatGPT encourage the user to check credible sources? (Yes/No)

- Example: "If you're unsure, consult official sources like the WHO or CDC."

---

### ** Output Format: **

Provide the classification results in the following structured format:

Coherence Appeals: [Yes/No]

Consensus Appeals: [Yes/No]

Credibility Appeals: [Yes/No]

Empathy Appeals: [Yes/No]

Encouragement to Verify: [Yes/No]

---

Content:

{entry}

---

**Now, analyze the conversation and provide structured output based on the defined criteria. **

"""

**Study Limitations and Possible Reasons for the Differences in Outcomes Between Issues**

This study has several limitations. The measures were not fully parallel across the two topics, which might constrain cross-issue comparisons. The flu vaccination misconception scale included only the evaluation of false/inaccurate statements, whereas the climate change misconception scale mixed false claims with scientifically accurate items. However, one could argue that reinforcing accurate beliefs may not be identical to refuting false claims. Thus, the fact that interactions with ChatGPT did not shift climate change misconceptions might be partly due to the artifact of the scale design where it might be more difficult to shift respondents’ agreement with mixed factual statements. Similarly, the flu vaccination attitude scale measured personal evaluations of getting vaccination (e.g., bad/good, harmful/beneficial), while the support for climate action scale combined personal worry about climate change with evaluation of current government action and desire for future government action. Future research might want to employ more aligned measurement structures for better cross-issue comparisons.

Beyond these measurement considerations, baseline climate change misconceptions were lower (M = 2.20) compared to flu vaccination related misconceptions (M = 2.93), leaving little room for change according to floor effects. In addition, ChatGPT used more consensus appeals and credibility appeals but fewer verification appeals and empathy appeals for climate change than for flu vaccination, which may also potentially account for the observed differences between the topics. Future research might want to examine issue topics that are more comparable in nature.

Another limitation of the study concerns the use of a pretest-posttest design rather than a posttest-only control group design. While a pretest-posttest design allows for more statistical power in our analysis with the current sample size, being asked the same questions a second time might sensitize respondents towards the purpose of the study and make them reconsider their initial answers to align with the information provided by ChatGPT. It is also possible that respondents might think more deeply about the topic during the interactions with ChatGPT, leading to changes in their answers even independent of ChatGPT’s contribution. As a result, future research might want to adopt a posttest only control group design that includes a baseline condition where participants interact with ChatGPT on an irrelevant topic or without ChatGPT exposure to better establish a more concrete causal relationship.

Also, while coherence appeals appeared in 100% of the transcripts, the instructions from the experiment explicitly directed users to evaluate statements about flu vaccination and climate change (including those widespread myths). Thus, it could be possible that AI might not generate these appeals as frequently in more naturalistic interactions.

In addition, respondents interacted with either GPT-3.5 or GPT-4. We did not record the specific version they used, but GPT-4 generally has higher reasoning capabilities and fewer hallucinations than GPT-3.5. As a result, it is possible that the positive outcomes observed might be more driven by a subset of users with access to the superior model. As generative AI technology continues to develop and as more advanced models become available featuring increased accuracy (reduced AI hallucinations), deeper reasoning abilities, and multimodal communication (combining texts, audios, and visuals), it is likely that using generative AI to reduce health related misconceptions may show more powerful effects as future research tests the effects from upgraded generative AI models.

Finally, we used a convenient university student sample in this pilot study. 217 students accessed the study and 149 completed it. It represents a dropout rate of about 31%. Given the nature of the study, those without access to ChatGPT and those with strong misconceptions on the topics in focus may have quit early. Future research might want to adopt a nationally representative sample to improve the generalizability of the findings across population groups.
